# DC-159a Shows Inhibitory Activity against DNA Gyrases of *Mycobacterium leprae*

**DOI:** 10.1371/journal.pntd.0005013

**Published:** 2016-09-28

**Authors:** Tomoyuki Yamaguchi, Kazumasa Yokoyama, Chie Nakajima, Yasuhiko Suzuki

**Affiliations:** 1 Division of Bioresources, Hokkaido University Research Center for Zoonosis Control, Sapporo, Japan; 2 Central Research Laboratory, Kissei Pharmaceutical Co., Ltd, Azumino, Japan; 3 Global Station for Zoonosis Control, Global Institution for Collaborative Research and Education (GI-CoRE), Hokkaido University, Sapporo, Japan; Fondation Raoul Follereau, FRANCE

## Abstract

**Background:**

Fluoroquinolones are a class of antibacterial agents used for leprosy treatment. Some new fluoroquinolones have been attracting interest due to their remarkable potency that is reportedly better than that of ofloxacin, the fluoroquinolone currently recommended for treatment of leprosy. For example, DC-159a, a recently developed 8-methoxy fluoroquinolone, has been found to be highly potent against various bacterial species. Nonetheless, the efficacy of DC-159a against *Mycobacterium leprae* is yet to be examined.

**Methodology/Principal Findings:**

To gather data that can support highly effective fluoroquinolones as candidates for new remedies for leprosy treatment, we conducted *in vitro* assays to assess and compare the inhibitory activities of DC-159a and two fluoroquinolones that are already known to be more effective against *M*. *leprae* than ofloxacin. The fluoroquinolone-inhibited DNA supercoiling assay using recombinant DNA gyrases of wild type and ofloxacin-resistant *M*. *leprae* revealed that inhibitory activities of DC-159a and sitafloxacin were at most 9.8- and 11.9-fold higher than moxifloxacin. Also the fluoroquinolone–mediated cleavage assay showed that potencies of those drugs were at most 13.5- and 9.8-fold higher than moxifloxacin. In addition, these two drugs retained their inhibitory activities even against DNA gyrases of ofloxacin-resistant *M*. *leprae*.

**Conclusions/Significance:**

The results indicated that DC-159a and sitafloxacin are more effective against wild type and mutant *M*. *leprae* DNA gyrases than moxifloxacin, suggesting that these antibacterial drugs can be good candidates that may supersede current fluoroquinolone remedies. DC-159a in particular is very promising because it is classified in a subgroup of fluoroquinolones that is known to be less likely to cause adverse effects. Our results implied that DC-159a is well worth further investigation to ascertain its *in vivo* effectiveness and clinical safety for humans.

## Author Summary

Leprosy is now recognized as a disease curable by chemotherapy. Although the number of leprosy cases has dramatically decreased as a result of multidrug therapy, there are still more than 210,000 new cases reported worldwide every year. Recurrence is a major concern in the control of leprosy. Relapses are usually considered to result from therapeutic failure due to inadequate or incomplete treatment. Thus, patient compliance with the planned course of medication is an important factor in the treatment outcome because multidrug therapy can take as long as up to 12 months. To improve patient compliance, it is imperative to develop and apply more potent drugs that can exert effect in a shorter period of treatment. In this study, focusing on the fluoroquinolone class of antimicrobials, we examined three powerful derivatives including newly developed DC-159a for their potencies against *Mycobacterium leprae*, the causative agent of leprosy. Our results indicate that the activity of DC-159a is sufficiently high and expected to surpass that of the currently used fluoroquinolone. This is the first strong evidence of the potential of this drug as a promising candidate for new leprosy remedies.

## Introduction

Leprosy is no longer an incurable disease. Since the introduction of Multidrug Therapy (MDT) by the World Health Organization in the 1980s, the number of registered leprosy cases has decreased dramatically. However, leprosy still remains a public health problem with more than 210,000 new cases every year mainly in Asian, Latin American, and African countries [1,2].

Fluoroquinolones (FQs) are considered to be important antibacterial drugs for treatment of leprosy. In current MDT regimens, ofloxacin is the FQ used for single skin lesion paucibacillary cases [3]. Although ofloxacin is adopted in MDT, it is not the most potent FQ. Bactericidal activity differs greatly among FQs. For example, moxifloxacin is known to be a more effective FQ against leprosy than ofloxacin [4–7], and its bactericidal activity is estimated to be equivalent to that of rifampicin, one of the first line drugs in MDT [4]. A study on human multibacillary leprosy cases demonstrated that moxifloxacin can kill leprosy bacilli with a single dose within days or weeks [5]. Sitafloxacin, another FQ, has also been found to be highly potent against *Mycobacterium leprae* in both *in vivo* and *in vitro* studies [8,9]. We previously assessed the inhibitory efficacies of FQs, including ofloxacin, moxifloxacin and sitafloxacin, and found that sitafloxacin was more effective than either ofloxacin or moxifloxacin [6,7]. Recently, DC-159a, a newly developed 8-methoxy FQ, has been reported to have high antimicrobial efficacy against various bacterial species including *M*. *tuberculosis* [10–12]. Although many studies have shown its potential as a remedy for bacterial infection, the efficacy of DC-159a against *M*. *leprae* has not been elucidated yet.

FQs interfere with DNA gyrase, a bacterial enzyme that plays an essential role in DNA replication and transcription [13,14]. DNA gyrase is a bacterial tetrameric enzyme composed of two subunits A (GyrA) and two subunits B (GyrB). Fluoroquinolone resistance can arise as a result of amino acid substitutions in the quinolone resistance-determining regions (QRDR) within the GyrA and GyrB subunits [15]. In *M*. *leprae*, only substitutions Gly to Cys at position 89 (Gly89Cys) and Ala to Val at position 91 (Ala91Val) in GyrA have been found to confer ofloxacin resistance in clinical strains [3,16]. In addition, we have experimentally proved that an amino acid substitution from Asp to Gly at position 95 (Asp95Gly) in GyrA, which is equivalent to the most frequently found amino acid substitution in FQ-resistant *M*. *tuberculosis* GyrA, also contributes to increased resistance to FQs in *M*. *leprae* [6].

Recurrence of leprosy is a major obstacle for control of the disease because relapse cases are more likely to be accompanied with resistance to drugs used in MDT, which limits the choice of anti-leprosy drugs [3,17–19]. Recurring cases are usually considered to result from therapeutic failure due to inadequate or incomplete treatment, and drug resistance can also be acquired at this time [20]. Thus, compliance with the planned course of medication is an important factor that can influence the treatment outcome because the recommended MDT can take as long as 12 months. To that end, introduction of FQs to MDT regimens that are more potent than ofloxacin, owing to their ability to clear *M*. *leprae* bacilli rapidly, would be expected to improve patient compliance by shortening the medication period. In this study, we focused on three powerful FQs, namely, moxifloxacin, sitafloxacin and DC-159a. In order to assess the potencies of these drugs as remedies for leprosy and to facilitate comparison between them, we conducted *in vitro* FQ-mediated assays using recombinant *M*. *leprae* DNA gyrases including wild type (WT) and mutants bearing amino acid substitutions Gly89Cys, Ala91Val and Asp95Gly.

## Materials and Methods

### Antibacterial agents

DC-159a and sitafloxacin were kindly provided by Daiichi-Sankyo Co., Ltd. (Tokyo, Japan). Moxifloxacin was purchased from LKT Laboratories, Inc. (St. Paul, MN). Ampicillin was purchased from Wako Pure Chemical Industries Ltd. (Osaka, Japan).

### Bacterial strains and expression plasmids

The Thai-53 strain of *M*. *leprae* [21], maintained at the Leprosy Research Center, National Institute of Infectious Diseases (Tokyo, Japan), was used to prepare *M*. *leprae* DNA. *Escherichia coli* strain TOP-10 (Thermo Fisher Scientific Inc.; Waltham, MA) was used for cloning. *E*. *coli* strains Rosetta-gami 2(DE3)pLysS and BL21(DE3)pLysS (Merck KGaA, Darmstadt, Germany) were used for protein expression. The plasmid vector pET-20b(+) (Merck KGaA) was used for the construction of expression plasmids. Relaxed and supercoiled pBR322 DNA (John Innes Enterprises Ltd.; Norwich, United Kingdom) were used for the DNA supercoiling assay and DNA cleavage assay.

### Construction of expression plasmids

DNA gyrase expression plasmids coding WT GyrA, GyrA with Ala91Val, GyrA with Asp95Gly and WT GyrB were constructed as described in our previous study [6]. The expression plasmid for GyrA with Gly89Cys was constructed in a similar way using primer pairs of k-45 (5ʹ-GGCATATGACTGATATCACGCTGCCACCAG-3ʹ) and k-58 (5ʹ-CGATGCGTCGCAGTGCGGATGG-3ʹ), and k-57 (5ʹ-CCATCCGCACTGCGACGCATCG-3ʹ) and k-46 (5ʹ-ATAACGCATCGCCGCGGGTGGGTCATTACC-3ʹ). The nucleotide sequence of GyrA gene with Gly89Cys in the plasmid was confirmed using a BigDye Terminator v3.1 cycle sequencing kit and an ABI Prism 3130xI genetic analyzer (Thermo Fisher Scientific Inc.).

### Expression and purification of recombinant DNA gyrase subunits

Recombinant DNA gyrase subunits were expressed and purified as previously described [6,22,23]. Briefly, each expression plasmid bearing either gyrA or gyrB of *M*. *leprae* was transformed in *E*. *coli* Rosetta-gami 2(DE3)pLysS or BL21(DE3)pLysS. The transformants were cultured in Luria-Bertani (LB) broth under ampicillin selection (100 μg/mL) up to the log phase. The expression of DNA gyrase was induced by the addition of 1 mM isopropyl-beta-D-thiogalactopyranoside (Wako Pure Chemical Industries Ltd., Osaka, Japan), and further incubation at 12 or 14°C for 16 to 24 h. The harvested *E*. *coli* were lysed by sonication (10 times for 40 s at output level 3 and 40% duty cycle with 40-s intervals) (Sonifier 250; Branson, Danbury, CT) and centrifugation (10,000× *g* for 30 min). The recombinant DNA gyrase subunits in the supernatants were purified by Ni-NTA Agarose (Thermo Fisher Scientific Inc.) column chromatography and dialyzed against DNA gyrase dilution buffer (50 mM Tris-HCl pH 7.5, 100 mM KCl, 2 mM DTT, 1 mM EDTA). The purified proteins were examined by sodium dodecyl sulfate-polyacrylamide gel electrophoresis (SDS-PAGE) with Prestained Protein Marker, Broad Range (7–175 kDa) (New England Biolab; Hitchin, UK).

### ATP-dependent DNA supercoiling assay

ATP-dependent DNA supercoiling assays were carried out as previously described [6,16,22–24]. Briefly, DNA supercoiling activities of the purified subunits were examined with a reaction mixture consisting of DNA gyrase reaction buffer, relaxed pBR322 DNA (180 ng), ATP (1 mM), and DNA gyrase subunits (30.0 ng of WT GyrA, GyrA with Gly89Cys, Ala91Val or Asp95Gly, 30.0 ng GyrA and 24.4 ng of WT GyrB) in a total volume of 18 μL. For the assays of DNA gyrase with Gly89Cys, 60.0 ng GyrA with Gly89Cys and 48.8 ng WT GyrB were also examined. The mixtures were incubated at 30°C for 1.5 h and the reaction was stopped by adding 4.5 μL of 5× dye mix (5% SDS, 25% glycerol, 0.25 mg/mL bromophenol blue). Next, 10 μL of each reaction mixture was subjected to electrophoresis with a 1% agarose 1× Tris-borate-EDTA buffer (TBE) gel. The agarose gel was stained with ethidium bromide (0.7 μg/mL).

### FQ-inhibited DNA supercoiling assay

The FQ-inhibited DNA supercoiling assay was based on the method described by Fisher and Pan [24]. Each assay was conducted in 18 μL of DNA gyrase reaction buffer (35 mM pH 7.5 Tris-HCl, 6 mM MgCl_2_, 1.8 mM spermidine, 24 mM KCl, 5 mM DTT, 0.36 mg/mL BSA, 6.5% w/v glycerol, 1mM ATP), with relaxed pBR322 DNA (180 ng), and DNA gyrase subunits. For the assays of WT DNA gyrase and mutant DNA gyrases with Ala91Val and Asp95Gly, 30.0 ng GyrA and 24.4 ng WT GyrB were mixed. For the assays of DNA gyrase with Gly89Cys, 60.0 ng GyrA with Gly89Cys and 48.8 ng WT GyrB were used instead. The reactions were continued at 30°C for 1.5 h and stopped by the addition of 4.5 μL of 5× dye mix (5% SDS, 25% glycerol, 0.25 mg/mL bromophenol blue). Next, 10 μL of each mixture was subjected to electrophoresis on 1% agarose 1× TBE gels and stained with ethidium bromide. To assess the inhibitory effects of FQs on DNA gyrases, the amount of DNA supercoiled in the reactions was quantified with ImageJ (http://rsbweb.nih.gov/ij) by determining the drug concentrations that inhibit DNA supercoiling by 50%, or half maximal inhibitory concentrations (IC_50_s), in the presence or absence of serial two-fold increases in the concentrations of DC-159a, sitafloxacin and moxifloxacin. Each assay was conducted three times to confirm its reproducibility.

### FQ-mediated DNA cleavage assay

The DNA cleavage assay was also based on the method by Fisher and Pan [24]. Each assay was carried out in 18 μL of the DNA gyrase reaction buffer with supercoiled pBR322 DNA (180 ng) and DNA gyrase subunits (the same concentrations as the ATP-dependent DNA supercoiling assays), and increasing concentrations of DC-159a, sitafloxacin and moxifloxacin. After 2-hour incubation at 30°C, the cleaving reactions were stopped by adding 2.7 μL of 2% SDS and 2.7 μL of proteinase K (1 mg/mL). Proteinase K reactions were continued for a further 30 min at 37°C, then stopped by the addition of 5.9 μL of 5× dye mix. Next, 10 μL of the reaction mixtures were electrophoresed on 0.8% agarose 1× TBE gels, and stained with ethidium bromide. To assess the FQ concentrations that convert 20% of input DNA to the linear form (CC_20_s), the amount of cleaved DNA was quantified with ImageJ. Each assay was conducted three times to confirm its reproducibility.

## Results

### Construction and purification of recombinant WT and mutant DNA gyrase subunits

**Fig 1:**
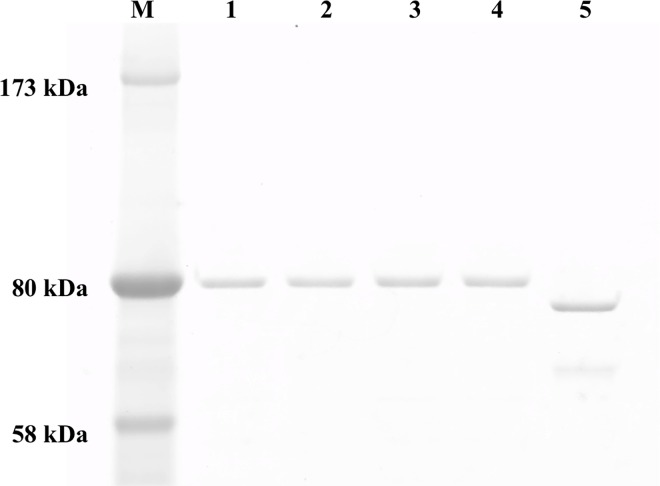
SDS-PAGE analysis of purified *M*. *leprae* DNA gyrase subunits. The His-tagged recombinant DNA gyrase subunits were expressed in *E*. *coli* and purified by Ni affinity column chromatography. One hundred ng of each purified subunit was loaded on a 5–20% gradient polyacrylamide gel. Lane M: Protein marker, lane 1: WT-GyrA, lane 2: Gly89Cys-GyrA, lane 3: Ala91Val-GyrA, lane 4: Asp95Gly-GyrA, lane 5: WT-GyrB.

The expression plasmids of WT GyrA, GyrA with Ala91Val, GyrA with Asp95Gly and WT GyrB previously constructed in this laboratory were used [6]. The DNA fragment including GyrA genes with Gly89Cys was amplified from the WT GyrA expression plasmid [6] and introduced into pET-20b (+). Recombinant subunits were expressed with C-terminal hexahistidine-tags (His-tags) for purification by Ni-NTA Agarose resin, as the His-tag does not interfere with the catalytic functions of GyrA and GyrB [6,7,16,22,23,25,26]. Expressed recombinant DNA gyrase subunits were purified as soluble His-tagged 80-kDa proteins of GyrA and 75-kDa proteins of GyrB. The purity of the recombinant proteins was confirmed by SDS-PAGE (Fig 1).

### ATP-dependent DNA supercoiling activities of DNA gyrases

**Fig 2:**
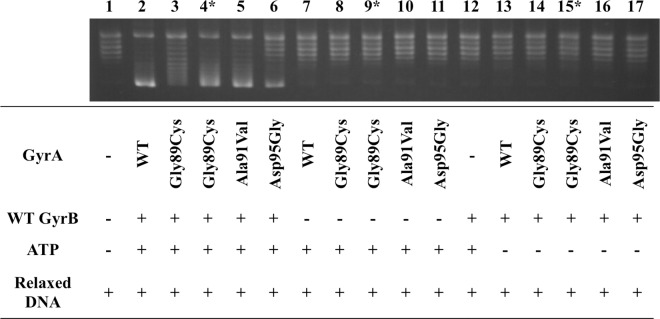
ATP-dependent DNA supercoiling assay. Supercoiling activities of DNA gyrases composed of WT-GyrA, Gly89Cys-GyrA, Ala91Val-GyrA or Asp95Gly-GyrA and WT-GyrB were confirmed. Relaxed pBR322 DNA was incubated with GyrA, GyrB, or both, of the subunits in the presence or absence of ATP. Lane 1: relaxed pBR322 DNA alone; lanes 2–6: relaxed pBR322 DNA, ATP, GyrA, and GyrB; lanes 7–11: relaxed pBR322 DNA, ATP, and GyrA; lane 12: relaxed pBR322 DNA, ATP, and GyrB; lane 13–17: relaxed pBR322 DNA, GyrA, and GyrB. ^*^ Twice the amount of DNA gyrase subunits was used in these assays.

Combinations of GyrA (WT or mutant with Gly89Cys, Ala91Val, or Asp95Gly) and WT-GyrB were examined for DNA supercoiling activities using relaxed pBR322 DNA as a substrate in the presence or absence of ATP (Fig 2). Relaxed DNA was supercoiled when GyrA, GyrB and ATP were all present; no supercoiling activity was observed in conditions lacking any of them.

### Half maximal inhibitory concentrations of FQs against WT and mutant DNA gyrases

**Fig 3:**
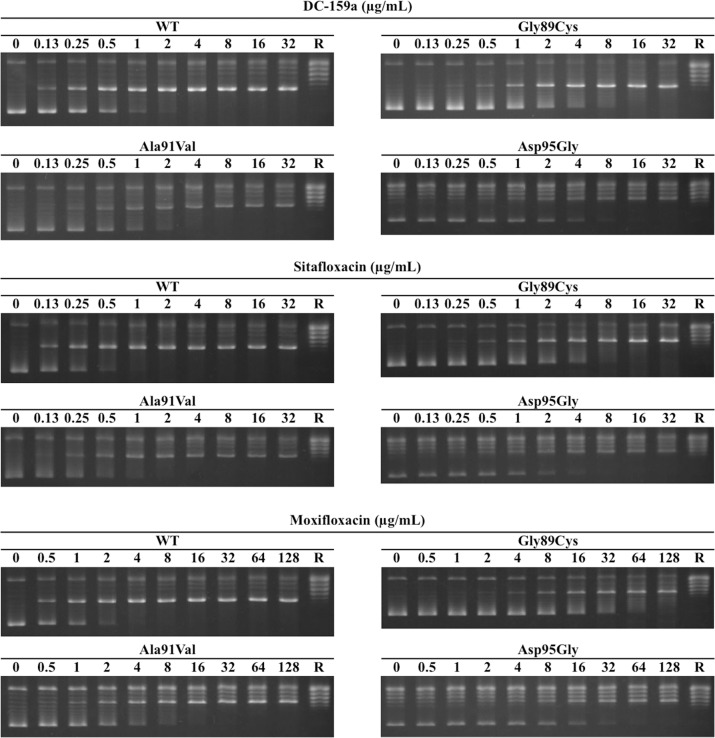
FQ-inhibited DNA supercoiling assay. Relaxed pBR322 DNA was mixed and incubated with GyrA, GyrB, ATP and FQs at the indicated concentrations. Each FQ was examined for its inhibitory activity against WT DNA gyrase and mutant DNA gyrases with Gly89Cys-GyrA (indicated as Gly89Cys), Ala91Val-GyrA (Ala91Val) and Asp95Gly-GyrA substitution (Asp95Gly). The lanes labeled as R indicate relaxed pBR322 DNA.

The IC_50_s were determined by the FQ-inhibited DNA supercoiling assays. Dose-dependent inhibition was observed in each combination of FQs and DNA gyrases (Fig 3). As shown in Table 1, the IC_50_s widely varied among the tested FQs. Both DC-159a and sitafloxacin showed much lower IC_50_s against every DNA gyrase than did moxifloxacin. Respective IC_50_s of DC-159a and sitafloxacin against WT DNA gyrase were 2.8- and 5.5-fold lower when compared with those of moxifloxacin, which were 9.8- and 11.9-fold lower against the DNA gyrase with Gly89Cys, 3.0-, 5.3-fold lower against the DNA gyrase with Ala91Val, and 4.4- and 6.4-fold lower against DNA gyrase with Asp95Gly. Fold changes of DC-159a and sitafloxacin between IC_50_s against the WT and the mutant DNA gyrases were at most 7.0 and 9.5, respectively, whereas that of moxifloxacin reached up to 20.5.

**Table 1:** IC_50_s of FQs against DNA gyrases of WT and mutant *M*. *leprae*.

Drug	IC_50_ (μg/mL)			
	WT	Gly89Cys	Ala91Val	Asp95Gly
DC-159a	0.4 ± 0.0	2.3 ± 0.3	0.7 ± 0.1	2.8 ± 0.1
Sitafloxacin	0.2 ± 0.0	1.9 ± 0.1	0.4 ± 0.1	1.9 ± 0.1
Moxifloxacin	1.1 ± 0.0	22.6 ± 2.9	2.1 ± 0.1	12.2 ± 1.3

### Concentrations of FQs that convert 20% of input DNA to the linear form against WT and mutant DNA gyrases

**Fig 4:**
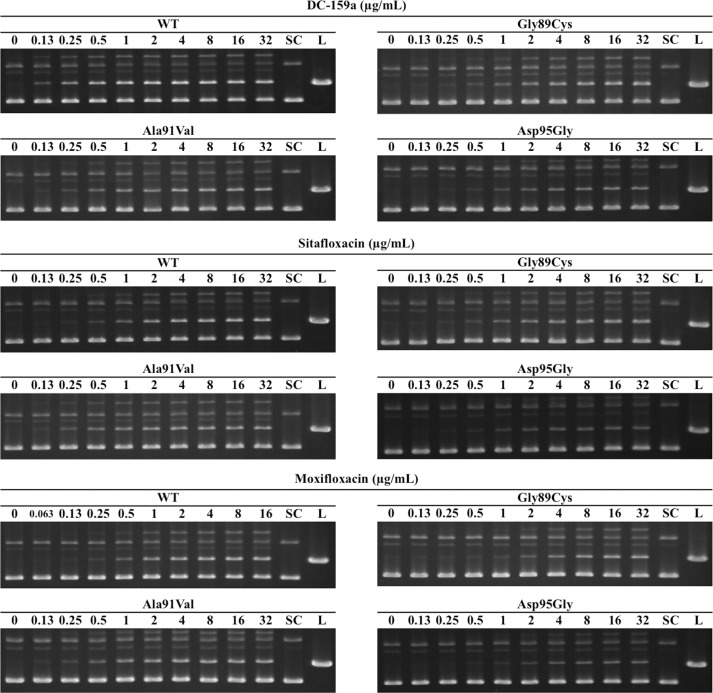
FQ-mediated DNA cleavage assay. Supercoiled pBR322 DNA was mixed and incubated with GyrA, GyrB, ATP and FQs at the indicated concentrations. Each FQ was examined for its cleaving activity against WT DNA gyrase and mutant DNA gyrases with Gly89Cys-GyrA (indicated as Gly89Cys), Ala91Val-GyrA (Ala91Val) and Asp95Gly-GyrA (Asp95Gly). The lanes labeled as SC and L indicate supercoiled and linearized pBR322 DNA, respectively.

The CC_20_s of the three FQs were determined by DNA cleavage assays. Dose-dependency of DNA cleavage is shown in Fig 4. The CC_20_s of all tested conditions are summarized in Table 2. The CC_20_s of DC-159a and sitafloxacin against all the tested DNA gyrases were lower than those of moxifloxacin. Respective CC_20_s of DC-159a against the WT and the mutant DNA gyrases with Gly89Cys, Ala91Val and Asp95Gly were 4.0-, 13.5-, 5.5- and 8.3-fold lower, and respective CC_20_s of sitafloxacin were 4.0-, 9.8-, 5.5- and 8.9-fold lower than those of moxifloxacin. Fold changes of DC-159a and sitafloxacin between CC_20_s against the WT and the mutant DNA gyrases were no more than 14.0 and 13.0, respectively, whereas that of moxifloxacin reached up to 29.0.

**Table 2:** CC_20_s of FQs against DNA gyrases of WT and mutant *M*. *leprae*.

Drug	CC_20_ (μg/mL)			
	WT	Gly89Cys	Ala91Val	Asp95Gly
DC-159a	0.1 ± 0.0	0.8 ± 0.1	0.4 ± 0.1	1.4 ± 0.3
Sitafloxacin	0.1 ± 0.0	1.1 ± 0.3	0.4 ± 0.1	1.3 ± 0.4
Moxifloxacin	0.4 ± 0.0	10.8 ± 2.2	2.2 ± 0.4	11.6 ± 1.9

## Discussion

In this study, we focused on FQs expected to have high potency against the DNA gyrases of WT and ofloxacin-resistant *M*. *leprae*. We examined the inhibitory activity of moxifloxacin, sitafloxacin and DC-159a by measuring their IC_50_s and CC_20_s using the FQ-inhibited DNA supercoiling assay and the FQ-mediated DNA cleavage assay using recombinant WT and mutant DNA gyrases.

Usually, potencies of antimicrobial agents against bacteria are evaluated and compared using minimum inhibitory concentrations (MICs) of the agents [27]. The MICs are defined as the lowest concentrations to inhibit visible growth of microorganisms, and are determined by conducting *in vitro* drug susceptibility tests, exposing target organisms directly to the agents. This parameter is also used to estimate clinical efficacies of antimicrobial drugs. However, determination of the MICs is not always possible. In the case of *M*. *leprae*, as this bacterium is yet to be cultured on any artificial media, MICs are not currently available. Thus, for FQ assessment, instead of MICs, ICs and CCs have been examined. In that regard, correlations between MICs and ICs or CCs have been reported in previous studies. For example, *M*. *tuberculosis*, which, similar to *M*. *leprae*, possesses DNA gyrase as a sole target of FQs, has high positive correlations between MIC and ICs or CCs [26]. Hence, for the present work we considered that *M*. *leprae* would also show this correlation and that IC_50_s and CC_20_s could be used for estimating bactericidal efficacies of FQs against *M*. *leprae*.

In the FQ-inhibited DNA supercoiling assay and the FQ-mediated cleavage assay, IC_50_s and CC_20_s of all tested FQs became lowest when they were examined for WT DNA gyrases. Interestingly, in both assays DC-159a and sitafloxacin always showed lower IC_50_s and CC_20_s than moxifloxacin. The lower level of these IC_50_s and CC_20_s indicated that DC-159a and sitafloxacin have higher potencies than moxifloxacin. In addition, IC_50_s and CC_20_s of DC-159a and sitafloxacin against the mutant DNA gyrases showed the lower fold changes from the values of the WT DNA gyrase when compared with that of moxifloxacin. That difference implied that DC-159a and sitafloxacin retained their inhibitory activities even against mutant DNA gyrases.

Compared with other types of DNA gyrases, twice the amounts of DNA gyrase subunits (60.0 ng of GyrA with Gly89Cys and 48.8 ng of WT-GyrB) were used for the assays of the DNA gyrase with Gly89Cys because IC_50_s and CC_20_s could not be measured at the same concentrations of DNA subunits. The extent of the effect of this substitution on IC_50_s of FQs has been previously estimated [16]. IC_50_s of FQs against the DNA gyrase with Gly89Cys were reported to be no lower than those against the DNA gyrase with Ala91Val [16]. This previously reported outcome indicates that Gly89Cys in GyrA can confer equal or higher resistance to FQs as Ala91Val. In the present study, we provide evidence that is in agreement with previous work. For example, we found IC_50_s of moxifloxacin against DNA gyrase with Gly89Cys to be more than 10 times higher than IC_50_s of moxifloxacin against DNA gyrase with Ala91Val. Even though it is likely that IC_50_s increased in the assays for DNA gyrase with Gly89Cys because twice the amount of DNA gyrase subunits was used, the large gap observed in IC_50_s between Gly89Cys and Ala91Val may not be solely due to differences in assay conditions.

Although moxifloxacin showed the highest IC_50_ and CC_20_ values for all types of DNA gyrase in the present work, it should be noted that moxifloxacin has been shown to be effective in leprosy treatment. For instance, in previous *in vitro* studies, moxifloxacin showed a much higher inhibitory effect on *M*. *leprae* DNA gyrase than did ofloxacin [6,7,16]. Moreover, a strong bactericidal activity of moxifloxacin was also reported in human cases of leprosy [5,28]. Therefore, the fact that in the present study DC-159a and sitafloxacin were shown to be more potent than moxifloxacin suggests that there is a strong likelihood they will be far more successful for treating leprosy than ofloxacin.

So far, only Gly89Cys and Ala91Val in GyrA have been found in clinical strains as amino acid substitutions that can confer ofloxacin resistance to *M*. *lepra*e, and a majority of ofloxacin-resistant *M*. *leprae* strains bear the latter [3]. In the present study, it was observed that the IC_50_s and CC_20_s of DC-159a and sitafloxacin against DNA gyrase with Ala91Val were the same or lower than those values for moxifloxacin against WT DNA gyrase. Taken together with the higher activity shown by moxifloxacin in comparison with ofloxacin, this outcome implies that DC-159a and sitafloxacin could be effective even against the majority of ofloxacin-resistant cases if the drugs could attain the same concentration in leprosy lesions as does moxifloxacin.

Sitafloxacin is already commercially available in Japan and Thailand. Its recommended dosage for bacterial infections is no more than 100 mg twice a day, whereas the other FQs are usually administered at a dosage of at least 200 mg once or twice a day [29,30]. Even at the relatively low dose of 100 mg twice a day, the pharmacokinetic and pharmacodynamic properties of sitafloxacin indicate that its efficacy against gram-positive or -negative bacteria is the same or better than that exerted by moxifloxacin and ofloxacin at their usual dose [30]. In addition, *in vivo* and *in vitro* studies have reported a good synergistic effect of sitafloxacin with rifampicin against *M*. *leprae* [8,9]. For these reasons, sitafloxacin seems to be a good option for the treatment of leprosy. However, there is a concern about its safe use in living bodies due to the presence of a chlorine atom at R_8_ in its structure and therefore, its administration remains controversial. For example, while some published articles reported no serious problems associated with sitafloxacin as long as it was administered at a clinically recommended dosage, others reported phototoxicity of this drug in *in vivo* experiments [29,31–35].

**Fig 5:**
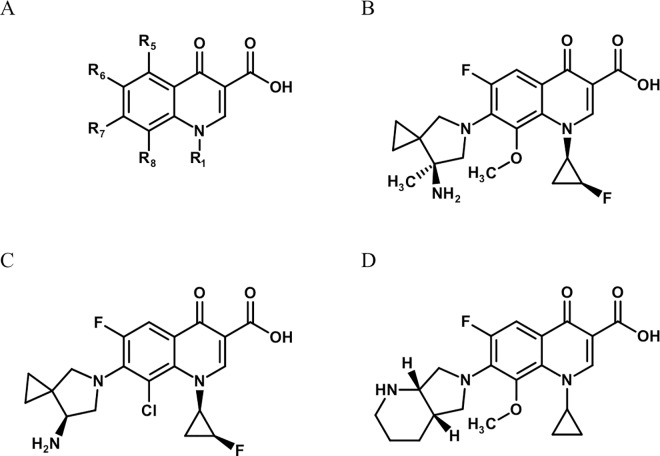
Structures of FQs tested in this study. (A) The basic structure of quinolones. (B) DC-159a. (C) sitafloxacin. (D) moxifloxacin.

In contrast, DC-159a is neither commercially available nor has it been assessed with *M*. *leprae* DNA gyrases yet. However, DC-159a has a similar structure to sitafloxacin (Fig 5). The noteworthy structural difference between these two drugs is at the R_8_ substituent. Indeed, DC-159a has a methoxy group at this position, whereas, as described above, sitafloxacin has a chlorine atom as the corresponding substituent. The R_8_ substituent has been considered to be responsible for adverse effects, especially phototoxicity and 8-methoxy FQs have been reported to have less phototoxicity than 8-halogenated FQs [31,36,37]. In fact, moxifloxacin, which has a methoxy group at the position, showed no phototoxicity in a mouse model [38,39]. Because FQs bring better therapeutic outcomes when they are administered at a higher dose and the adverse effects are mostly dose-dependent, this characteristic may be crucial [36,40]. In this respect, DC-159a seems to have an advantage. It can then be expected that this drug will have a lower frequency of adverse effects than 8-halogenated FQs in patients with skin lesions when it is administered at high doses to achieve success in treatment of ofloxacin-resistant leprosy.

In conclusion, we found that the inhibitory activities of DC-159a and sitafloxacin are sufficient and these drugs are much more effective against *M*. *leprae* DNA gyrases with any reported mutations related to FQ resistance than moxifloxacin. Moreover, we showed that these drugs possess strong inhibitory effects even against the mutant DNA gyrases of most ofloxacin-resistant strains. DC-159a in particular seems to be a very promising candidate that may supersede the current FQ remedies because its structural characteristics suggest a reduced likelihood of adverse effects. However, current *in vivo* data for DC-159a including its distribution to skin lesions and its adverse effects in humans are still scarce. Our findings provide strong evidence to warrant further investigation to assess the effectiveness of DC-159a in clinical leprosy cases.
